# Equations for the Magnetic Field Produced by One or More Rectangular Loops of Wire in the Same Plane

**DOI:** 10.6028/jres.105.045

**Published:** 2000-08-01

**Authors:** Martin Misakian

**Affiliations:** National Institute of Standards and Technology, Gaithersburg, MD 20899-8113

**Keywords:** equations, electronic article surveillance systems, magnetic field, metal detector, rectangular coils

## Abstract

Beginning with expressions for the vector potential, the equations for calculating the magnetic flux density from up to three rectangular loops of wire in the same plane are derived. The geometry considered is the same as that found in some walk-through metal detectors and electronic article surveillance systems. Equations for more or fewer loops can be determined by inspection. A computer program for performing the magnetic field calculation is provided in an appendix.

## 1. Introduction

The expression for the magnetic flux density from a single rectangular loop of wire of many turns can be found in text books and various publications [1–3]. The rectangular geometry is convenient, in part, because the expressions for the three spatial components of the flux density are in closed form. Single square coils have been used for calibration of extremely low frequency magnetic field meters for applications that require uncertainties of a few percent [2]. Multiple rectangular loops with a common axis have found applications in a number of fields, including biological exposure systems for in vivo and in vitro studies [3,4]. It is also noteworthy that a square Helmholtz coil produces a greater volume of nearly uniform magnetic field than a circular Helmholtz coil of comparable dimensions [5]. This paper develops expressions for the magnetic flux density produced by three rectangular loops of wire that lie in the same plane, i.e., loops that are not co-axial. The geometry is similar to that used in some walk-through metal detectors and electronic article surveillance systems. By inspection, the expressions for more or fewer loops are easily determined. We consider static and time varying fields that are quasi-static. In the latter case, the wavelength *λ* of the time varying field is much greater than any dimension or distance of interest. For example, a 1 MHz alternating field (*λ* ≈ 300 m) is well approximated as being quasi-static a few meters or less from loops of comparable dimensions. The quasi-static condition allows us to solve the static field problem first and, with negligible error, introduce the time dependence as a multiplicative factor, e.g., the direct current in the field equations could be replaced with an alternating current. The field equations are for rectangular loops with a single turn of wire. The magnetic flux density for loops with more than one turn are found by multiplying the equations by the appropriate number of turns.

## 2. Field Equations

We follow the development of Weber [1] by first considering the vector potential for a rectangular loop of wire in the *x-y* plane, *A_x_* and *A_y_*, and then calculating the vector components of the magnetic flux density using the relations

$$B_{x}=-\frac{\partial A_{y}}{\partial z},\;B_{y}=\frac{\partial A_{x}}{\partial z},\;B_{z}=\frac{\partial A_{y}}{\partial x}-\frac{\partial A_{x}}{\partial y}.$$ (1)

**Fig. 1:**
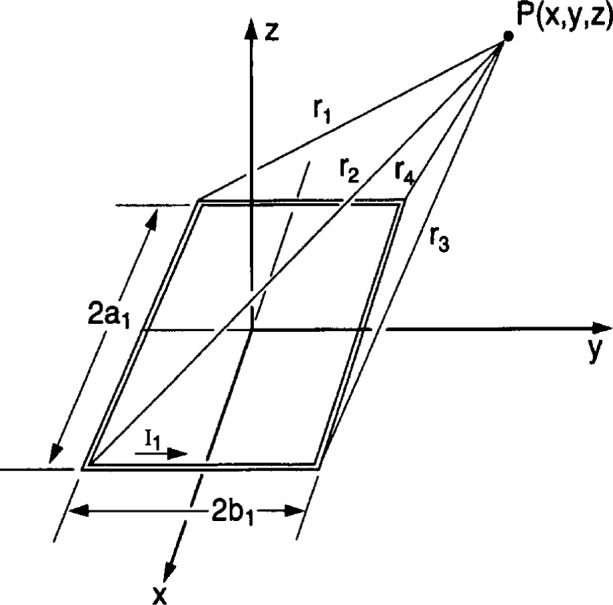
Geometry for a single rectangular loop of wire in the *x-y* plane. The magnetic flux density is evaluated at point P(*x*, *y*, *z*).

For a single rectangular loop of wire of negligible wire cross section, designated as loop 1, with side dimensions 2*a*_1_ by 2*b*_1_ as shown in Fig. 1, the components of the vector potential are [1]

$$A_{x1}=\frac{\mu_{0}I_{1}}{4\pi }\;\ln\;\left[\frac{\left(r_{1}+a_{1}+x\right)}{\left(r_{2}-a_{1}+x\right)}\cdot \frac{\left(r_{3}-a_{1}+x\right)}{\left(r_{4}+a_{1}+x\right)}\right],$$ (2)

and

$$A_{y1}=\frac{\mu_{0}I_{1}}{4\pi }\;\ln\;\left[\frac{\left(r_{2}+b_{1}+y\right)}{\left(r_{3}-b_{1}+y\right)}\cdot \frac{\left(r_{4}-b_{1}+y\right)}{\left(r_{1}+b_{1}+y\right)}\right],$$ (3)

where *μ*_0_ is the magnetic constant (also called the magnetic permeability of vacuum), and *I*_1_ is the current in the loop.

The parameters *r*_1_, *r*_2_, *r*_3_, and *r*_4_ are the distances from the corners of the loop to the point P(*x*, *y*, *z*) where the magnetic flux density will be evaluated (see below and Fig. 1).

The *z*-component of the magnetic flux density at P(*x*, *y*, *z*) is

$$B_{z1}=\frac{\mu_{0}I_{1}}{4\pi }\sum_{a=1}^{4}\left[\frac{{\left(-1\right)}^{\alpha }d_{\alpha }}{r_{\alpha }\left[r_{\alpha }+{\left(-1\right)}^{\alpha +1}C_{\alpha }\right]}-\frac{C_{\alpha }}{r_{\alpha }\left[r_{\alpha }+d_{\alpha }\right]}\right],$$ (4)

where

$$\begin{array}{l} \begin{array}{cc} C_{1}=-C_{4}=a_{1}+x & d_{1}=d_{2}=y+b_{1}\\ C_{2}=-C_{3}=a_{1}-x & d_{3}=d_{4}=y-b_{1} \end{array}\\ \;r_{1}=\sqrt{{\left(a_{1}+x\right)}^{2}+{\left(y+b_{1}\right)}^{2}+z^{2}}\\ \;r_{2}=\sqrt{{\left(a_{1}-x\right)}^{2}+{\left(y+b_{1}\right)}^{2}+z^{2}}\\ \;r_{3}=\sqrt{{\left(a_{1}-x\right)}^{2}+{\left(y-b_{1}\right)}^{2}+z^{2}}\\ \;r_{4}=\sqrt{{\left(a_{1}+x\right)}^{2}+{\left(y-b_{1}\right)}^{2}+z^{2}}. \end{array}$$

Equation (4) is equivalent to that given in Ref. [1], but perhaps in a more convenient form for writing a computer program to calculate the magnetic flux density.

From Eqs. (1) to (3), the expressions for the *x*- and *y*-components of the magnetic flux density can be readily derived and are

$$B_{x1}=\frac{\mu_{0}I_{1}}{4\pi }\sum_{\alpha =1}^{4}\left[\frac{{\left(-1\right)}^{\alpha +1}z}{r_{\alpha }\left[r_{\alpha }+d_{\alpha }\right]}\right],$$ (5)

and

$$B_{y1}=\frac{\mu_{0}I_{1}}{4\pi }\sum_{\alpha =1}^{4}\left[\frac{{\left(-1\right)}^{\alpha +1}z}{r_{\alpha }\left[r_{\alpha }+{\left(-1\right)}^{\alpha +1}C_{\alpha }\right]}\right].$$ (6)

**Fig. 2:**
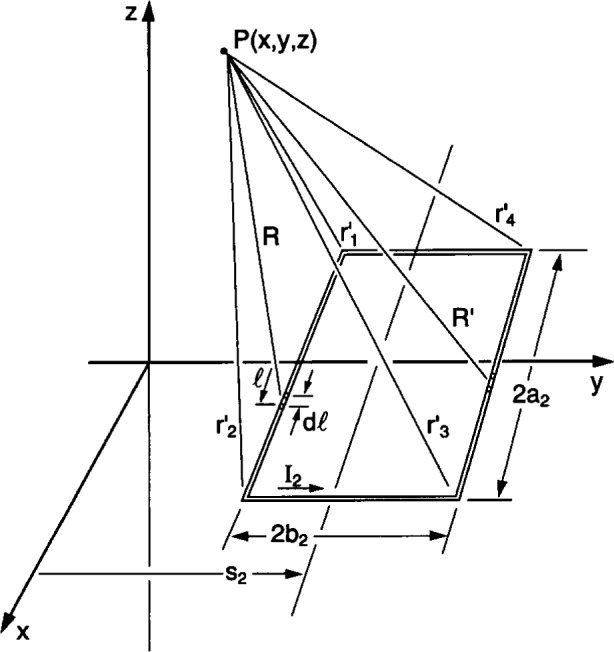
Geometry for a second rectangular loop of wire in *x-y* plane. The point P(*x*, *y*, *z*) coincides with that in Fig. 1 (note that the scales of Figs. 1 and 2 are not the same).

The *x*-component of the vector potential for a second loop of wire of side dimensions 2*a*_2_ by 2*b*_2_ that is displaced from the origin by a distance *s*_2_ and bisected by the *y*-axis (see Fig. 2) is given by [1]

$$A_{x2}=\frac{\mu_{0}I_{2}}{4\pi }\left[\int_{l=-a_{2}}^{l=a_{2}}\frac{dl}{R}+\int_{l=a_{2}}^{l=-a_{2}}\frac{dl}{R^{\prime }}\right]$$ (7)

where

$$\begin{array}{l} R=\sqrt{{\left(x-l\right)}^{2}+{\left(y-s_{2}+b_{2}\right)}^{2}+z^{2}}\\ R^{\prime }=\sqrt{{\left(x-l\right)}^{2}+{\left(y-s_{2}-b_{2}\right)}^{2}+z^{2}}, \end{array}$$

and *I*_2_ is the current in loop 2.

The integrals can be solved using elementary methods and yield

$$A_{x2}=\frac{\mu_{0}I_{1}}{4\pi }\;\ln\;\left[\frac{\left({r^{\prime }}_{1}+a_{2}+x\right)}{\left({r^{\prime }}_{2}-a_{2}+x\right)}\cdot \frac{\left({r^{\prime }}_{3}-a_{2}+x\right)}{\left({r^{\prime }}_{4}+a_{2}+x\right)}\right],$$ (8)

where ${r^{\prime }}_{1}$, ${r^{\prime }}_{2}$, ${r^{\prime }}_{3}$, and ${r^{\prime }}_{4}$ are the distances from the corners of loop 2 to the point P(*x*, *y*, *z*) where the magnetic flux density will be evaluated (see below).

The expression for *A_y_*_2_ can be similarly determined and is given by

$$A_{y2}=\frac{\mu_{0}I_{1}}{4\pi }\;\ln\;\left[\frac{\left({r^{\prime }}_{2}+b_{2}+y-s_{2}\right)}{\left({r^{\prime }}_{3}-b_{2}+y-s_{2}\right)}\cdot \frac{\left({r^{\prime }}_{4}-b_{2}+y-s_{2}\right)}{\left({r^{\prime }}_{1}+b_{2}+y-s_{2}\right)}\right].$$ (9)

Taking the appropriate derivatives of Eqs. (8) and (9), the expression for the *z*-component of the magnetic flux density at P(*x*, *y*, *z*) associated with loop 2 is

$$B_{z2}=\frac{\mu_{0}I_{2}}{4\pi }\sum_{\alpha =1}^{4}\left[\frac{{\left(-1\right)}^{\alpha }{d^{\prime }}_{\alpha }}{{r^{\prime }}_{\alpha }\left[{r^{\prime }}_{\alpha }+{\left(-1\right)}^{\alpha +1}{C^{\prime }}_{\alpha }\right]}-\frac{{C^{\prime }}_{\alpha }}{{r^{\prime }}_{\alpha }\left({r^{\prime }}_{\alpha }+{d^{\prime }}_{\alpha }\right)}\right],$$ (10)

where

$$\begin{array}{l} \begin{array}{cc} {C^{\prime }}_{1}=-{C^{\prime }}_{4}=a_{2}+x & {d^{\prime }}_{1}={d^{\prime }}_{2}=y+b_{2}-s_{2}\\ {C^{\prime }}_{2}=-{C^{\prime }}_{3}=a_{2}-x & {d^{\prime }}_{3}={d^{\prime }}_{4}=y-b_{2}-s_{2} \end{array}\\ \begin{array}{c} \;{r^{\prime }}_{1}=\sqrt{{\left(a_{2}+x\right)}^{2}+{\left(y+b_{2}-s_{2}\right)}^{2}+z^{2}}\\ \;{r^{\prime }}_{2}=\sqrt{{\left(a_{2}-x\right)}^{2}+{\left(y+b_{2}-s_{2}\right)}^{2}+z^{2}}\\ \;{r^{\prime }}_{3}=\sqrt{{\left(a_{2}-x\right)}^{2}+{\left(y-b_{2}-s_{2}\right)}^{2}+z^{2}}\\ \;{r^{\prime }}_{4}=\sqrt{{\left(a_{2}+x\right)}^{2}+{\left(y-b_{2}-s_{2}\right)}^{2}+z^{2}}. \end{array} \end{array}$$

From Eqs. (1), (8), and (9), the *x*- and *y*-components of the magnetic flux density due to loop 2 are

$$B_{x2}=\frac{\mu_{0}I_{2}}{4\pi }\sum_{\alpha =1}^{4}\left[\frac{{\left(-1\right)}^{\alpha +1}z}{{r^{\prime }}_{\alpha }\left[{r^{\prime }}_{\alpha }+{d^{\prime }}_{\alpha }\right]}\right]$$ (11)

and

$$B_{y2}=\frac{\mu_{0}I_{2}}{4\pi }\sum_{\alpha =1}^{4}\left[\frac{{\left(-1\right)}^{\alpha +1}z}{{r^{\prime }}_{\alpha }\left[{r^{\prime }}_{\alpha }+{\left(-1\right)}^{\alpha +1}{C^{\prime }}_{\alpha }\right]}\right].$$ (12)

The equations for the flux density components at P(*x*, *y*, *z*) from a third rectangular loop with side dimensions 2*a*_3_ by 2*b*_3_, displaced from the origin by a distance *s*_3_ and bisected by the *y*-axis follow by inspection. That is

$$B_{z3}=\frac{\mu_{0}I_{3}}{4\pi }\sum_{a=1}^{4}\left[\frac{{\left(-1\right)}^{\alpha }{d^{\prime \prime }}_{\alpha }}{{r^{\prime \prime }}_{\alpha }\left[{r^{\prime \prime }}_{\alpha }+{\left(-1\right)}^{\alpha +1}{C^{\prime \prime }}_{\alpha }\right]}-\frac{{C^{\prime \prime }}_{\alpha }}{{r^{\prime \prime }}_{\alpha }\left[{r^{\prime \prime }}_{\alpha }+{d^{\prime \prime }}_{\alpha }\right]}\right].$$ (13)

where

$$\begin{array}{l} \begin{array}{cc} {C^{\prime \prime }}_{1}=-{C^{\prime \prime }}_{4}=a_{3}+x & {d^{\prime \prime }}_{1}={d^{\prime \prime }}_{2}=y+b_{3}-s_{3}\\ {C^{\prime \prime }}_{2}=-{C^{\prime \prime }}_{3}=a_{3}-x & {d^{\prime \prime }}_{3}={d^{\prime \prime }}_{4}=y-b_{3}-s_{3} \end{array}\\ \begin{array}{c} \;{r^{\prime \prime }}_{1}=\sqrt{{\left(a_{3}+x\right)}^{2}+{\left(y+b_{3}-s_{3}\right)}^{2}+z^{2}}\\ \;{r^{\prime \prime }}_{2}=\sqrt{{\left(a_{3}-x\right)}^{2}+{\left(y+b_{3}-s_{3}\right)}^{2}+z^{2}}\\ \;{r^{\prime \prime }}_{3}=\sqrt{{\left(a_{3}-x\right)}^{2}+{\left(y-b_{3}-s_{3}\right)}^{2}+z^{2}}\\ \;{r^{\prime \prime }}_{4}=\sqrt{{\left(a_{3}+x\right)}^{2}+{\left(y-b_{3}-s_{3}\right)}^{2}+z^{2}}, \end{array} \end{array}$$

and *I*_3_ is the current in loop 3.

The *x*- and *y*-components of the magnetic flux density due to loop 3 are

$$B_{x3}=\frac{\mu_{0}I_{3}}{4\pi }\sum_{\alpha =1}^{4}\left[\frac{{\left(-1\right)}^{\alpha +1}z}{{r^{\prime \prime }}_{\alpha }\left[{r^{\prime \prime }}_{\alpha }+{d^{\prime \prime }}_{\alpha }\right]}\right]$$ (14)

and

$$B_{y3}=\frac{\mu_{0}I_{3}}{4\pi }\sum_{\alpha =1}^{4}\left[\frac{{\left(-1\right)}^{\alpha +1}z}{{r^{\prime \prime }}_{\alpha }\left[{r^{\prime \prime }}_{\alpha }+{\left(-1\right)}^{\alpha +1}{C^{\prime \prime }}_{\alpha }\right]}\right].$$ (15)

**Fig. 3:**
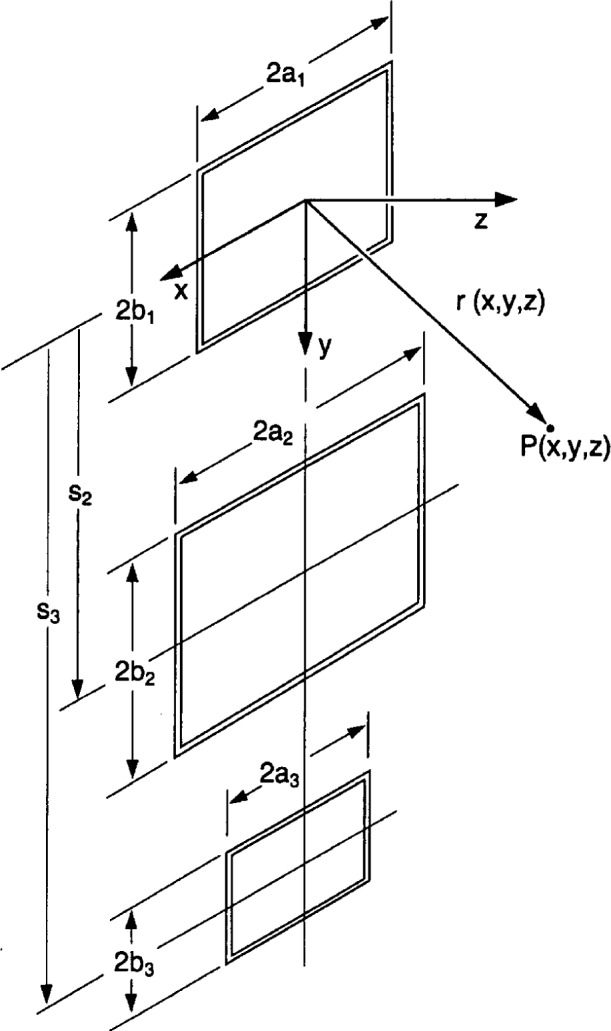
Geometry for three rectangular loops of wire in a vertical plane. The origin of the coordinate system is at the center of loop 1.

The spatial components of the magnetic flux density at P(*x*, *y*, *z*) due to all three loops (Fig. 3) are found by summing the respective contributions from each loop, i.e.,

$$\begin{array}{c} B_{zT}=B_{z1}+B_{z2}+B_{z3}\\ B_{xT}=B_{x1}+B_{x2}+B_{x3}\\ B_{yT}=B_{y1}+B_{y2}+B_{y3}. \end{array}$$ (16)

For direct currents in the loops, the direction of the magnetic flux density will remain fixed and is described by the vector

$$B=B_{xT}i+B_{yT}j+B_{zT}k,$$ (17)

where ***i***, ***j***, and ***k*** are unit vectors along the *x*, *y*, and *z* directions, respectively. The magnitude of the magnetic flux density vector will also be constant and equal to

$$|B|=\sqrt{B_{xT}^{2}+B_{yT}^{2}+B_{zT}^{2}}.$$ (18)

For alternating currents in the loops that are in phase, for example *I*_1_sin(*ωt*), *I*_2_sin(*ωt*), and *I*_3_sin(*ωt*), the magnetic flux density is described by the vector

$$B=\left[B_{xT}i+B_{yT}j+B_{zT}k\right]\sin\left(\omega t\right).$$ (19)

where *I*_1_, *I*_2_, and *I*_3_ are current amplitudes, *ω* is the angular frequency, and *t* is the time. The flux density is said to be linearly polarized because of its oscillatory motion along a straight line. The magnitude of the vector will be time dependent and equal to

$$|\sqrt{B_{xT}^{2}+B_{yT}^{2}+B_{xT}^{2}}\sin\left(\omega t\right)|.$$

If the alternating currents in the various loops are not in phase, the magnetic flux density vector will rotate and the point of the vector will, in general, trace an ellipse [6]. The magnitude *and* direction of the magnetic flux density at a given point in space will change as a function of time. For this case, the flux density is said to be elliptically polarized.

As a convenience to the reader, a program for calculating the static magnetic flux density from three coils in the *x-y* plane as shown in Fig. 3 is provided in Appendix A.

## 3. Appendix A. Program for Calibrating the Magnetic Flux Density

The Quick Basic^1^ program below calculates the magnetic flux density at a point *x*, *y*, *z* specified in the input statement for three rectangular loops of single turn wire with side dimensions 2*a*_1_, 2*b*_1_, 2*a*_2_, 2*b*_2_, 2*a*_3_, and 2*b*_3_. The loops are separated by distances *s*_2_ and *s*_3_, and are bisected by the *y*-axis. The origin of the coordinate system is at the center of loop 1. Figure 3 shows the geometry of the three loops and a point P(*x*, *y*, *z*) where the magnetic flux density is calculated. The program assumes that *I*_1_ = *I*_2_ = *I*_3_ = 1 A of direct current and that the current is in the counter clockwise direction as one views the current looking down on the *x-y* plane (e.g., see Fig. 1). All dimensions are in meters and the magnetic flux density will be in units of 10^−7^ T. For example, with 2*a*_1_, 2*b*_1_, 2*a*_2_, 2*b*_2_, 2*a*_3_, and 2*b*_3_ equal to 1.0 m, 0.5 m, 2.0 m, 1 m, 0.5 m, and 0.3 m, respectively, separated by distances *s*_2_ = 1.5 m and *s*_3_ = 2.5 m, the magnitude of the magnetic flux density |***B***| at *x* = 0.75 m, *y* = 0.75 m, and *z* = 1 m is 0.95170 × 10^−7^ T.

REM PROGRAM TO CALCULATE B-FIELD FROM THREE RECTANGULAR SINGE TURN COILS WITH REM SIDE DIMENSIONS 2A1 X 2B1, 2A2 X 2B2, AND 2A3 X 2B3, AND SEPARATED BY DISTANCES REM S2 AND S3, RESPECTIVELY. ALL THREE LOOPS ARE IN THE X-Y PLANE AND THE REM Y-AXIS BISECTS EACH RECTANGLE; SEE FIG. 3.
DEFDBL A-H, K-Z
CLS
DIM C(4), D(4), P(4), Q(4), T(4), R(4)
DIM CP(4), DP(4), PP(4),QP(4), TP(4), RP(4)
DIM CPP(4), DPP(4), PPP(4), QPP(4), TPP(4), RPP(4)
PRINT “ENTER: A1, B1, A2, B2, A3, B3, S2, S3, X, Y, Z”
INPUT A1, B1, A2, B2, A3, B3, S2, S3, X, Y, Z
REM TERMS FOR LOOP 1
C(1) = A1 + X
C(2) = A1 – X
C(3) = −C(2)
C(4) = −C(1)
D(1) = B1 + Y
D(2) = D(1)
D(3) = Y − B1
D(4) = D(3)
R(1) = SQR(C(1)^2 + D(1)^2 + Z^2)
R(2) = SQR(C(2)^2 + D(1)^2 + Z^2)
R(3) = SQR(C(2)^2 + (B1 − Y)^2 + Z^2)
R(4) = SQR(C(1)^2 + (B1 − Y)^2 + Z^2)
REM CORRESPONDING TERMS FOR LOOP 2
CP(1) = A2 + X
CP(2) = A2 – X
CP(3) = −CP(2)
CP(4) = −CP(1)
DP(1) = Y + B2 − S2
DP(2) = DP(1)
DP(3) = Y − B2 − S2
DP(4) = DP(3)
RP(1) = SQR(CP(1)^2 + DP(1)^2 + Z^2)
RP(2) = SQR(CP(2)^2 + DP(1)^2 + Z^2)
RP(3) = SQR(CP(2)^2 + DP(3)^2 + Z^2)
RP(4) = SQR(CP(1)^2 + DP(4)^2 + Z^2)
REM CORRESPONDING TERMS FOR LOOP 3
CPP(1) = A3 + X
CPP(2) = A3 – X
CPP(3) = −CPP(2)
CPP(4) = −CPP(1)
DPP(1) = Y + B3 − S3
DPP(2) = DPP(1)
DPP(3) = Y − B3 − S3
DPP(4) = DPP(3)
RPP(1) = SQR(CPP(1)^2 + DPP(1)^2 + Z^2)
RPP(2) = SQR(CPP(2)^2 + DPP(1)^2 + Z^2)
RPP(3) = SQR(CPP(2)^2 + DPP(3)^2 + Z^2)
RPP(4) = SQR(CPP(1)^2 + DPP(4)^2 + Z^2)
REM CALCULATE X COMPONENT OF LOOP 1 (BX1)
FOR J = 1 TO 4
P(J) = (Z*(−1)^(J+1))/((R(J) + D(J))*R(J))
NEXT J
BX1 = 0
FOR I = 1 TO 4
BX1 = BX1 + P(I)
NEXT I
PRINT “BXFIELD1 =”; BX1
REM CALCULATE Y COMPONENT OF LOOP 1 (BY1)
FOR J = 1 TO 4
P(J) = (Z*(−1)^(J+1))/((R(J) + ((−1)^(J +1))*C(J))*R(J))
NEXT J
BY1 = 0
FOR I = 1 TO 4
BY1 = BY1 + P(I)
NEXT I
PRINT “BYFIELD1 =”; BY1
REM CALCULATE Z COMPONENT OF LOOP 1 (BZ1)
FOR J = 1 TO 4
P(J) = ((−1)^J)*D(J)/(R(J)*(R(J) + ((−1)^(J + 1))*C(J)))
Q(J) = C(J)/(R(J)*(R(J) + D(J)))
T(J) = P(J) + Q(J)
NEXT J
BZ1 = 0
FOR I = 1 TO 4
BZ1 = BZ1 + T(I)
NEXT I
PRINT “BZFIELD1 =”; BZ1
PRINT
REM CORRESPONDING TERMS FOR LOOP 2; BEGIN WITH X COMPONENT OF LOOP 2 (BX2)
FOR J = 1 TO 4 PP(J) = (Z*(−1)^(J+1))/((RP(J) + DP(J))*RP(J))
NEXT J
BX2 = 0
FOR I = 1 TO 4
BX2 = BX2 + PP(I)
NEXT I
PRINT “BXFIELD2 =”; BX2
REM CALCULATE Y COMPONENT OF LOOP 2 (BY2)
FOR J = 1 TO 4
PP(J) = (Z*(−1)^(J+1))/((RP(J) + ((−1)^(J +1))*CP(J))*RP(J))
NEXT J
BY2 = 0
FOR I = 1 TO 4
BY2 = BY2 + PP(I)
NEXT I
PRINT “BYFIELD2 =”; BY2
REM CALCULATE Z COMPONENT OF LOOP 2 (BZ2)
FOR J = 1 TO 4
PP(J) = ((−1)^J)*DP(J)/(RP(J)*(RP(J) + ((−1)^(J + 1))*CP(J)))
QP(J) = −CP(J)/(RP(J)*(RP(J) + DP(J)))
TP(J) = PP(J) + QP(J)
NEXT J
BZ2 = 0
FOR I = 1 TO 4
BZ2 = BZ2 + TP(I)
NEXT I
PRINT “BZFIELD2 =”; BZ2
PRINT
REM CORRESPONDING TERMS FOR LOOP 3; BEGIN WITH X COMPONENT OF LOOP 3 (BX3)
FOR J = 1 TO 4
PPP(J) = (Z*(−1)^(J+1))/((RPP(J) + DPP(J))*RPP(J))
NEXT J
BX3 = 0
FOR I = 1 TO 4
BX3 = BX3 + PPP(I)
NEXT I PRINT “BXFIELD3 =”; BX3
REM CALCULATE Y COMPONENT OF LOOP 3 (BY3)
FOR J = 1 TO 4
PPP(J) = (Z*(−1)^(J+1))/((RPP(J) + ((−1)^(J +1))*CPP(J))*RPP(J))
NEXT J
BY3 = 0
FOR I = 1 TO 4
BY3 = BY3 + PPP(I)
NEXT I
PRINT “BYFIELD3 =”; BY3
REM CALCULATE Z COMPONENT OF LOOP 3 (BZ3)
FOR J = 1 TO 4
PPP(J) = ((−1)^J)*DPP(J)/(RPP(J)*(RPP(J) + ((−1)^(J + 1))*CPP(J)))
QPP(J) = −CPP(J)/(RPP(J)*(RPP(J) + DPP(J)))
TPP(J) = PPP(J) + QPP(J)
NEXT J
BZ3 = 0
FOR I = 1 TO 4
BZ3 = BZ3 + TPP(I)
NEXT I
PRINT “BZFIELD3 =”; BZ3
PRINT
REM COMBINE SPATIAL COMPONENTS OF B-FIELD FROM LOOPS 1, 2, AND 3
BXT = BX1 + BX2 + BX3
BYT = BY1 + BY2 + BY3
BZT = BZ1 + BZ2 + BZ3
REM MAGNITUDE OF B VECTOR
BMAG = SQR(BXT^2 + BYT^2 + BZT^2)
PRINT “BXTOTAL =”; BXT; “BYTOTAL =”; BYT; “BZTOTAL =”; BZT
PRINT “MAGNITUDE OF B =”; BMAG
PRINT “X =”; X; “Y =”; Y; “Z =”; Z
END
